# Clinicopathological profile and outcomes of pediatric glomerular diseases: A tertiary care experience

**DOI:** 10.12669/pjms.42.9.13794

**Published:** 2026-09

**Authors:** Bilquis Naeem, Lubna Aman, Hina Fatima, Shazia Soomro

**Affiliations:** 1 Bilquis Naeem, MBBS, FCPS Pediatric Medicine, FCPS Pediatric Nephrology, Assistant Professor, Pediatric Medicine, Departments of Pediatric Nephrology, National Institute of Child Health (NICH), Jinnah Sindh Medical University (JSMU), Karachi, Pakistan; 2 Lubna Aman, MBBS, FCPS Pediatric Medicine, FCPS Pediatric Nephrology, Assistant Professor, Pediatric Nephrology, Pediatric Medical Unit, National Institute of Child Health (NICH), Jinnah Sindh Medical University (JSMU), Karachi, Pakistan; 3 Hina Fatima, MBBS, FCPS Pediatric Medicine, FCPS Pediatric Nephrology Assistant Professor, Pediatric Nephrology, Pakistan Health Research Centre, National Institute of Child Health (NICH), Jinnah Sindh Medical University (JSMU), Karachi, Pakistan; 4 Shazia Soomro, MBBS, FCPS Pediatric Medicine Assistant Professor, Pediatric Medicine, National Institute of Child Health (NICH), Jinnah Sindh Medical University (JSMU), Karachi, Pakistan

**Keywords:** Biopsy, Glomerular diseases, Indications, Renal outcome

## Abstract

**Background & Objective:**

Glomerular diseases in children are significant cause of morbidity and mortality, causing significant burden on health care system. Data regarding the outcomes of paediatric patients with glomerular diseases in association with clinicopathological findings is critical to understand the management plan and prognosis. To determine clinical spectrum, histopathological findings and outcome of children with glomerular disease who underwent kidney biopsy.

**Methodology:**

Cross sectional retrospective study conducted at nephrology department of National Institute of Child Health from January 2016 to December 2020. All children aged one to 18 years who underwent percutaneous kidney biopsy were included. Data recorded from the files including demographics, clinical presentations, diagnosis, histopathological findings, post biopsy complications and their outcome at last follow-up were noted. Data were analyzed using SPSS version 23.

**Results:**

Seventy children with mean age of 6.85±3.54 were studied. Males were predominant (56.9%). Most common presentation was edema (95.8%) followed by oliguria (77.7%) and hypertension (44.4%). Most common indication for kidney biopsy was steroid resistant nephrotic syndrome (SRNS 56.9%) followed by rapid progressive glomerulonephritis (RPGN16.7%). Two most common histopathological findings were focal segmental glomerulosclerosis (FSGS 33%) and minimal change disease (MCD 26%). Biopsy complications were observed in 21%. Outcome of our study showed that majority (75.7%), had preserved renal functions whereas, 5.6 % developed chronic kidney disease.

**Conclusion:**

Common presentations were edema, oliguria and hypertension. SRNS and RPGN were common indications for kidney biopsy. FSGS and MCD were common histopathological findings. Outcome was normal kidney functions in majority and few developed chronic kidney disease.

## INTRODUCTION

Glomerular diseases contribute significant morbidity and are major concern in children kidney health. Idiopathic nephrotic syndrome (NS) is a prevalent kidney disorder in children, with different patients characteristics over various regions of the world. Kidney biopsy though not routinely performed in children but plays a pivotal role in both diagnosis and prognosis of glomerular diseases.^1^ Glomerular diseases can be primary or secondary, occurring in the setting of systemic autoimmune diseases, infections, familial, drugs, or malignancy, affecting all ages. The pathological diagnosis of kidney lesions is important for treatment decision and outcome of patients with glomerulonephritis.^2^ So, pathologic diagnosis demands kidney biopsy, an invasive but very important diagnostic tool.^3^

With the advent of light microscopy (LM), immunoflourescent (IM) and electron microscopy (EM) an accurate tissue diagnosis is now possible. Common indications of biopsy include steroid resistant nephrotic syndrome(SRNS), steroid dependent nephrotic syndrome (SDNS) after cyclophosphamide use, IgA vasculitis nephritis, Lupus nephritis and rapidly progressive glomerulonephritis (RPGN).^4^ In contrast to adults, steroid sensitive nephrotic syndrome (SSNS) does not require biopsy since minimal change disease (MCD) is the most likely histopathological diagnosis and thus the number of children requiring kidney biopsy has been reduced after historic international study of kidney disease in children (ISKDC).^5^,^6^ Complications are uncommon in children undergoing ultrasound guided renal biopsy and thus kidney biopsy is considered as safe procedure.^7^

Diseases specific renal outcomes over five years follow up after biopsy are reported in various studies.^8^-^11^ Focal segmental glomerulosclerosis (FSGS) and MCD are the two most common histopathological diagnosis seen in renal biopsies among SRNS.^12^ Chronic kidney disease(CKD) has been reported as a long -term outcome in these children with SRNS and underlying diagnosis of FSGS.^13^

To our best knowledge little work is done on outcome of the patients who underwent renal biopsies in our setup, so, the aim of our study was to determine the clinical characteristics, indications for biopsy, their histopathological pattern, post-biopsy complications and kidney outcome of patients over two to five years. This data will help to better understand the spectrum of glomerular diseases and their outcome and thus may help in better clinical counseling.

## METHODOLOGY

A retrospective cross-sectional study was conducted in the department of pediatric nephrology at National Institute of Child Health, Karachi.

### Ethical approval:

It was obtained from Institutional Ethical Review Board (IERB EX-11/2023; dated April 19, 2023). Individual consent from patient or parents is not required in this retrospective study.

### Inclusion Criteria:

All children who underwent kidney biopsy for diagnosis of glomerular disease from the January 2016 to December 2020 were included.

Percutaneous ultrasound guided left kidney biopsy was performed using semi-automated gun under sedation analgesia according to the institutional protocol which includes parental informed consent and pre biopsy relevant blood workup. Data was collected from medical records and included demographics, clinical presentations (edema, hypertension, hematuria, and oliguria), laboratory data (serum creatinine, serum albumin, serum cholesterol, urine analysis and serology; ANA, anti -Ds DNA, C3 and C4 levels), indications for biopsy and complications (peri-renal hematoma, local infection, hematuria and pain) observed during study period.

Kidney outcome of patients were also recorded and categorized as normal kidney function, development of chronic kidney disease (CKD), end stage kidney disease (ESKD) required kidney support therapy, lost to follow-up and mortality during the study period.

### Operational definition:

### SRNS:

Steroid resistance nephrotic syndrome was defined as failure to achieve remission after full dose of prednisolone (2mg/kg/day) for four to six weeks.^14^

**Fig.1:**
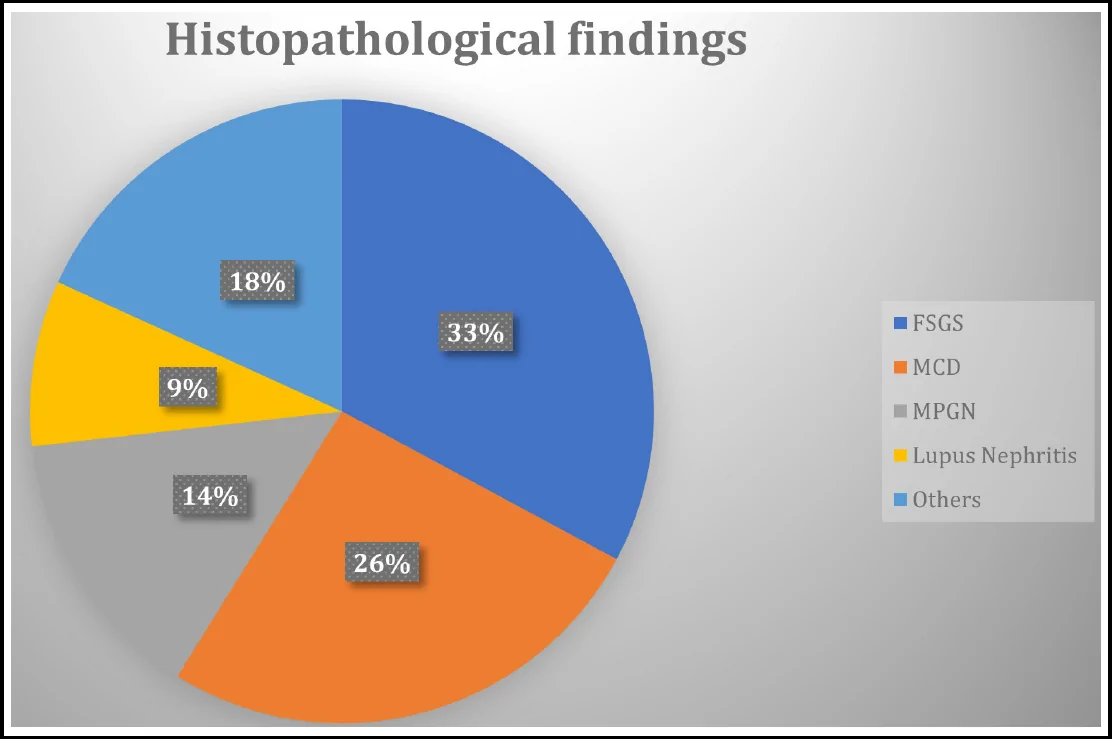
Histopathological diagnosis in children who underwent kidney biopsy.

### SDNS:

Steroid dependent nephrotic syndrome is defined as two consecutive relapses while on treatment with prednisone (full dose or tapering dose) or within 15 days of stopping of treatment.^14^

### FRNS:

Frequent relapsing nephrotic syndrome was defined as two or more relapses within six months of disease onset or four relapses over 12 months in disease course.^14^

### Lupus Nephritis:

Patient diagnosed as systemic lupus erythematosus with proteinuria(dipstick protein >2 or spot urine protein to creatinine ratio (PCR)>0.5 with or without urinary active sediments(dysmorphic red blood cells (RBC), RBC and or granular casts).^14^

### IgA vasculitis Nephritis:

Patient diagnosed as Henoch-Schönlein purpura (HSP) with persistent proteinuria (spot urine PCR >0.2) in an early morning sample or impaired kidney function (eGFR <80ml/min/1.73m^2^).^15^

### RPGN:

Rapidly progressive glomerulonephritis was defined by features of glomerulonephritis (hematuria, proteinuria, edema, HTN, oliguria) and rapid decline in renal function over days or weeks.^16^

### Normal renal function:

Estimated glomerular filtration rate (eGFR) more 90ml/min/1.73m^2^ calculated by Schwartz formula.^17^

### Chronic Kidney disease (CKD):

Patients with eGFR less than 90ml/min/1.73m^2^ persisted for more than three months.

### End stage kidney disease:

Patients who required for kidney support therapy (dialysis or kidney SRNS, FRNS, and SDNS) were treated with daily calcineurin inhibitors (CNIs), such as cyclosporine or tacrolimus, along with angiotensin-converting enzyme inhibitors (ACEi) or angiotensin receptor blockers (ARBs), in addition to corticosteroids (prednisolone 1 mg/kg on alternate days). Patients with FRNS and SDNS were those who continued to relapse despite treatment with levamisole and/or oral cyclophosphamide.

Children presenting with rapidly progressive glomerulonephritis (RPGN) were treated with pulse methylprednisolone for five days, followed by tapering doses of steroids over six months. Monthly pulse cyclophosphamide (500 mg/m²) was administered in cases where biopsy showed Class III or IV lupus nephritis or crescentic glomerulonephritis with ≥50% glomerular involvement. Patients with diabetic nephropathy were managed with ACE inhibitors or ARBs along with adjustment of insulin dosage. Treatment of IgA vasculitis included ACE inhibitors or ARBs in combination with corticosteroids

### Statistical analysis:

Data was analyzed using SPSS version 23. Categorical variables including gender distribution, signs & symptoms, diagnostic findings, outcome etc. are described as frequencies or percentages while continuous variables including age, weight and height are reported as mean±SD.

## RESULTS

We reviewed record of 70 children who underwent renal biopsy during the study period. The mean age was 6.85 years ± 3.54 and age of onset was 5.46 ± 3.62 years. Predominant patients were males (n 40,57%) and females were (n 30,42.2%) with male to female ratio of 1.3:1. Mean weight was 20.6 ± 9.76 kg and mean height was 110.53 ± 23.36 cm. The clinical features of these children include edema (n 69,95.8%) oliguria(n 56,77.8%), hypertension (n 32,44.4%), gross hematuria(n 13,18.1%), arthritis (n 8,11.1%), purpura (n 4, 5.6%), convulsions (n 2,8%). Clinical diagnosis was made as Primary nephrotic syndrome (n 49,68.1%), rapidly progressive glomerulonephritis (n 11,15.3%), secondary nephrotic syndrome (n8,12.5%), persistent non-nephrotic range proteinuria (n1,1.4%), recurrent hematuria (n1,1.4%) and congenital nephrotic Syndrome (n1,1.4%). Most common clinical indication of renal biopsy was steroid resistance (N 41,56%) patients followed by rapidly progressive glomerulonephritis 12(16.7%) as shown in Table-I. Hematuria was the most common complication observed in (N 13, 18.5%), other complications included perinephric hematoma (N 7, 10%), site infection (N1,1.4%) and local pain (N 1,1.4%).

**Table-I:** Clinical indications of kidney biopsy in children studied(N=70).

Clinical feature	Number (percentage)
SRNS	41(56%)
SDNS	3(4.2%)
FRNS	4(5.6%)
RPGN	12(16.7%)
Lupus nephritis	6(8.3%)
IgAV	2(2.8%)
IgA Nephropathy	1(1.4%)
Diabetic Nephropathy	1(1.4%)

SRNS: Steroid Resistant nephrotic Syndrome, SDNS: Steriod dependent Nephrotic Syndrome, FRNS: Frequent relapsing Nephrotic syndrome, RPGN: Rapidly progresssive glomerulonephritis, IgAV: IgA vasculitis.

Among the kidney histopathological (HP) diagnosis, FSGS (N 23, 32.8%) was the most common. Others HP diagnoses were MCD(N18, 25.7%), Membranoproliferative glomerulonephritis (MPGN N 10, 14.2%), Lupus nephritis (N 6,8.5%), Membranous nephropathy (N 2,2.8 %), Mesangial proliferative GN(N 2, 2.8%), Diabetic nephropathy(N1,1.4%), Amyloidosis(N 2, 2.8%) Hemolytic uremic syndrome (N1, 1.40%) Post infectious GN(N1,1.4%) IgA nephropathy(N1,1.4%), Tubulointerstitial nephritis (N1, 1.4%) and IgA vasculitis nephritis (N 2, 2.8%).

Various treatment options offered were oral corticosteroid alone (N 2,2.8%), calcineurin inhibitors(CNI) like cyclosporine or tacrolimus) with steroids (N32,44.4%), Angiotensin converting enzyme blocker (ACE)/Angiotensin receptor blocker (ARB) alone (N10,13.9%), Calcineurin Inhibitors(CNI) and Mycophenolate Mofetil (MMF) (N1,1.4%), steroids , Cyclophosamide (CYP) and ACE or ARB (N11,15.3%), steroids, CNI and ACE or ARB (N2, 2.8%). Four patients (5.6%) with diagnosis of RPGN underwent hemodialysis. Table-III

**Table-II:** Post biopsy complications in children (n=22).

Perinephric hematoma	7 (10%)
Gross hematuria	13 (18.5%)
Biopsy site infection	1 (1.4%)
Biopsy site pain	1 (1.4%)

**Table-III:** Treatment Options (n=70).

Oral corticosteriods	2 (2.8%)
CNI + corticosteriods	38 (54.4%)
ACE or ARBs	12(17.1%)
CNI + MMF	1 (1.4%)
CYP+ACE/ARB+ corticosteriods	11(15.7%)
CNI+ACE/ARB+ corticosteriods	2(2.8%)
Hemodialysis	4 (5.6%)

Follow up of children with glomerular disease ranged from two to five years. Majority of patients (n53, 75.71%) continued to have preserved kidney functions whereas 4 (5.6%) patients with histopathological diagnosis of focal segmental glomerulosclerosis development CKD. Two patients (2.8%) were lost to follow up. Seven patients (9.7%) expired. Focal segmental glomerulonephritis (N5), Amyloidosis (N1) and Lupus Nephritis (N1) were underlying histopathological diagnosis in children who expired during study period.

## DISCUSSION

This study describes the spectrum of histopathological diagnosis in children with various glomerular diseases at single center. In our study, there was a male predominance(57%) which is consistent with existing literature where it ranged from 56.3% - 62.9% .^18^-^20^

The mean age of presentation was 6.85±3.54 years. Comparable results are shown by Kanjilal S et al. where mean age was 6.77+3.42 years^21^ and Priyadarshini et al who reported mean age of 5.94 years (range four months – 14 years).^15^ Males were predominant in our study (57%) like Priyadarshini et al.^15^ (62.9%). However, male to female ratio was equal in Kanjilal S et al.^21^ study.

The most common indication of renal biopsy was SRNS (56%) followed by RPGN (16,7%) in our study at our center. This is consistent with previous studies by Kanjilal S et al.^21^, in which SRNS was the most common indication for renal biopsy. The study conducted by Saca et al.^19^ also demonstrate SRNS as most common indication for renal biopsy in children. A regional study by A Sadaf et al.^22^ also reported SRNS as common indication. Another study reported unexplained kidney failure as the most common clinical indication followed by nephrotic syndrome.^23^

We found FSGS as a most common histopathological pattern in (33%) followed by MCD (26%) in our study. Other studies have reported similar findings like study by Safdar et al.^24^ (FSGS 40.76%), Momtaz et al.^25^ (FSGS 32.7%), and Kanjilal et al.^21^ who reported FSGS as predominant histopathological pattern (45.9%). Post renal biopsy hematoma was found in seven patients(10%) in our study, which is lower as compared to a study by C pettit^7^ 28% done by interventional radiologist and 42% by pediatric nephrologist. The duration of study and number of study participant may express such diverse picture. Which could be very small or minimal as exact dimensions need to be evaluated.

In study by Alhasan K et al.^5^ they found eight patients (2.5%) with Alport syndrome confirmed on renal biopsy but in our study, we didn’t find any this may be our sample size is smaller 70 as compare to their study that is 326 between 1998-2017. This study reveals that patients undergoing renal biopsy primarily present with primary nephrotic syndrome, particularly steroid resistant and rapidly progressive forms. Similar indications were found in study at Nepal by Amatya M et al.^26^ and Wannous H et al.^27^ These contrasts to study at Hong Kong where systemic diseases were the most common indications.^19^ this could be variation in indications in developed and developing countries. Regarding outcome of patients in this study, overall, 53 patients (75.71%) had normal renal function, 4 (5.6%) patients developed chronic kidney disease, 4 (5.6%) underwent dialysis. This is more or less similar to study a study from India^20^ in which 83.5% of patients had a favorable course (69.2% and 4.4% developed CKD.

We found a significant mortality (10%) and among them five had FSGS as underlying diagnosis. Our mortality is higher than the mortality rate (5.9%) reported by Momtaz et al.^25^, which was contributed by ESKD and complications of SRNS course. The better outcomes observed in the present study appear to be due to a higher proportion of late resistance.

### Limitations:

The retrospective nature of the study and the small sample size are the major limitations of our research. Assessing disease outcomes, especially across different histopathological subtypes, is challenging due to various entities of glomerular diseases with underlying variety of histopathological diagnosis and varying treatment protocols. A prospective study with a larger sample size, which compares outcomes based on specific histopathological findings, would provide more robust data and better insights into the disease progression and prognosis.

## CONCLUSION

This study adds valuable insights into clinicopathological profile and outcome of various glomerular diseases in children at a tertiary care setting. The majority of children requiring renal biopsy were diagnosed clinically as steroid resistant nephrotic syndrome with underlying histopathology of focal segmental glomerulosclerosis. While most children maintained preserved renal function, a subset of children developed chronic kidney disease (CKD), this might be due to short term follow up and need long term follow up.
